# Aldosterone Negatively Regulates Nrf2 Activity: An Additional Mechanism Contributing to Oxidative Stress and Vascular Dysfunction by Aldosterone

**DOI:** 10.3390/ijms22116154

**Published:** 2021-06-07

**Authors:** Daniel Rodrigues, Tiago J. Costa, Josiane F. Silva, José Teles de Oliveira Neto, Juliano V. Alves, Aline G. Fedoce, Rafael Menezes Costa, Rita C. Tostes

**Affiliations:** 1 Department of Pharmacology, Ribeirao Preto Medical School, University of Sao Paulo, 14049-900 Ribeirao Preto, Brazil; daniel2.rodrigues@usp.br (D.R.); tjcosta@usp.br (T.J.C.); jozibio@gmail.com (J.F.S.); telesneto@usp.br (J.T.d.O.N.); juliano.vilela@yahoo.com (J.V.A.); alinefedoce@usp.br (A.G.F.); 2 Health Sciences Special Academic Unit, Federal University of Jatai, 75804-020 Jataí, Brazil; rafael.menezess@yahoo.com.br

**Keywords:** aldosterone, vascular dysfunction, oxidative stress, Nrf2

## Abstract

High levels of aldosterone (Aldo) trigger oxidative stress and vascular dysfunction independent of effects on blood pressure. We sought to determine whether Aldo disrupts Nrf2 signaling, the main transcriptional factor involved in antioxidant responses that aggravate cell injury. Thoracic aorta from male C57Bl/6J mice and cultured human endothelial cells (EA.hy926) were stimulated with Aldo (100 nM) in the presence of tiron [reactive oxygen species (ROS) scavenger, eplerenone [mineralocorticoid receptor (MR) antagonist], and L-sulforaphane (SFN; Nrf2 activator). Thoracic aortas were also isolated from mice infused with Aldo (600 μg/kg per day) for 14 days. Aldo decreased endothelium-dependent vasorelaxation and increased ROS generation, effects prevented by tiron and MR blockade. Pharmacological activation of Nrf2 with SFN abrogated Aldo-induced vascular dysfunction and ROS generation. In EA.hy926 cells, Aldo increased ROS generation, which was prevented by eplerenone, tiron, and SFN. At short times, Aldo-induced ROS generation was linked to increased Nrf2 activation. However, after three hours, Aldo decreased the nuclear accumulation of Nrf2. Increased Keap1 protein expression, but not activation of p38 MAPK, was linked to Aldo-induced reduced Nrf2 activity. Arteries from Aldo-infused mice also exhibited decreased nuclear Nrf2 and increased Keap1 expression. Our findings suggest that Aldo reduces vascular Nrf2 transcriptional activity by Keap1-dependent mechanisms, contributing to mineralocorticoid-induced vascular dysfunction.

## 1. Introduction

Increased aldosterone (Aldo) plasma concentrations are reported in subjects with congestive heart failure [1], resistant hypertension [2], type 2 diabetes mellitus [3], obesity [4], and metabolic syndrome [5]. Aldo excess has a plethora of deleterious effects: it increases blood pressure, produces oxidative stress, inflammation, and fibrosis, effects that cause end-organ damage in cardiovascular and metabolic diseases [6].

Mineralocorticoid receptors (MR) play a significant role in Aldo signaling. In renal tubular cells, Aldo binding to MR and translocation of the Aldo/MR complex to the nucleus stimulates the expression and membrane translocation of epithelial sodium channels (ENaC), which increases Na+ reabsorption by the kidneys [7]. Aldo has extrarenal effects and activates MR in endothelial cells, vascular smooth muscle cells (VSMCs), cardiomyocytes, immune cells, and adipocytes, contributing to the complexity of the pathophysiology of cardiovascular diseases [6]. Clinical and experimental data clearly show the beneficial effects of MR antagonism, highlighting the translational importance of the Aldo–MR axis in cardiovascular and metabolic diseases [8,9].

Redox signaling, or signaling processes whereby an oxidant generates a biologic response, is essential in many cellular processes, including cell proliferation, differentiation, cell cycle, and angiogenesis [10,11]. Major mediators of redox signaling, reactive oxygen species (ROS), are produced primarily by the mitochondria and NAD(P)H oxidase enzymes (NOXes) [12], and are finely regulated by components of the antioxidant system. In conditions where cellular ROS production becomes exacerbated and the antioxidant system is unable to inactivate ROS, cells enter into oxidative stress status. Oxidative stress leads to a dysregulated phenotype that contributes to the pathophysiology of many conditions, including hypertension, diabetes, obesity, and atherosclerosis [11,13].

Oxidative stress modifies both the structure and function of many key proteins in vascular biology. The superoxide anion (O_2_^−^), for example, interacts with nitric oxide (NO), generates peroxynitrite (ONOO^−^), and increases vascular tone [12]. Superoxide anion is rapidly converted by superoxide dismutase-1 (SOD1) to hydrogen peroxide (H_2_O_2_), another major redox messenger in vascular biology. Hydrogen peroxide increases the oxidation of proteins that control vascular smooth muscle cells’ relaxation and contraction [10].

Aldo has a potent pro-oxidant effect. In VSMCs and mesangial cells, Aldo induces oxidative stress via NOX activation and non-receptor tyrosine kinase, c-Src-dependent mechanisms [14]. Aldo promotes cerebral vascular dysfunction and endothelial dysfunction, which is mediated by increased ROS generation [15]. Furthermore, Aldo-induced ROS generation also contributes to neonatal rat cardiac myocytes apoptosis and renal injury [16,17], supporting that ROS generation is key to Aldo-induced vascular injury.

Antioxidant defense systems are activated to counterbalance cellular oxidative stress and restore redox homeostasis. The nuclear factor erythroid 2-related factor 2 (Nrf2) is the major redox-sensitive transcriptional factor regulating gene expression of many antioxidant and detoxifying enzymes [18,19,20]. In the basal state, Nrf2 is repressed and kept in the cytoplasm by the Keap1/Cul3 complex proteins, which lead Nrf2-–Keap1/Cul3 to ubiquitination and degradation in the ubiquitin–proteasome system. In oxidative stress conditions, or in the presence of pharmacological Nrf2 activators, Nrf2 nuclear translocation is facilitated by the oxidation of cysteine (Cys) residues in Keap1. Keap1 dissociates from Nrf2, which translocates into the nucleus and binds to the antioxidant response element (ARE) of various genes, starting an antioxidant response [21,22].

Many compounds and small molecules interfere with Nrf2 activation. L-sulforaphane (SFN), a known Nrf2 activator, prevents angiotensin II-induced VSMCs migration through Nrf2 upregulation and ROS/NOX axis suppression [23]. Furthermore, SFN attenuates cardiac oxidative damage in type 2 diabetic mice by increasing Nrf2 transcriptional activity [24]. Cardiac hypertrophy and oxidative damage induced by chronic exposure to angiotensin II are prevented by SFN treatment, which upregulates Nrf2 transcriptional activity and inhibits Akt/GSK-3β signaling [25]. Accordingly, Nrf2 siRNA in cultured cardiomyocytes blunts the protective effects of SFN [23,26], reinforcing the importance of impaired Nrf2 signaling and activity in pathological conditions. Mice treated for 14 days with bardoxolone methyl (BM), another Nrf2 activator, exhibit increased renal expression of Nrf2 and Nrf2-regulated genes, decreased ROS generation, and less tubulointerstitial injury in response to Aldo and salt-induced renal injury [27].

Although Nrf2 activation protects from Aldo—salt-dependent renal damage [27]—little is known about the interplay between Aldo and Nrf2 in the vasculature. Here we determine whether Aldo interferes with Nrf2 activation and the mechanisms leading to this effect. We also addressed whether Nrf2 activation prevents oxidative damage and vascular dysfunction induced by Aldo. Considering that Aldo plays an essential role in controlling the vascular tone, has pro-oxidant properties, and that ROS contribute to vascular dysfunction, we tested the hypothesis that high levels of the mineralocorticoid not only increase ROS generation but reduce Nrf2 activity, contributing to Aldo-induced vascular dysfunction.

## 2. Results

### 2.1. Aldosterone, via Mineralocorticoid Receptor (MR) Activation, Induces ROS Generation and Vascular Dysfunction

**Figure 1:**
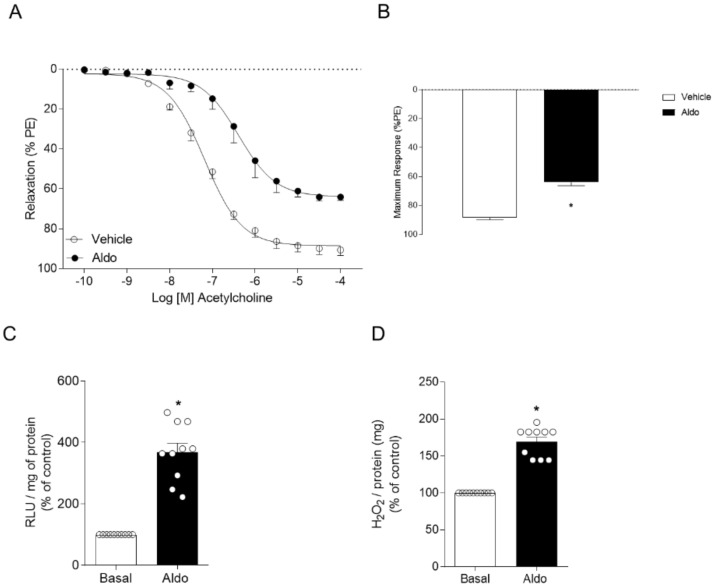

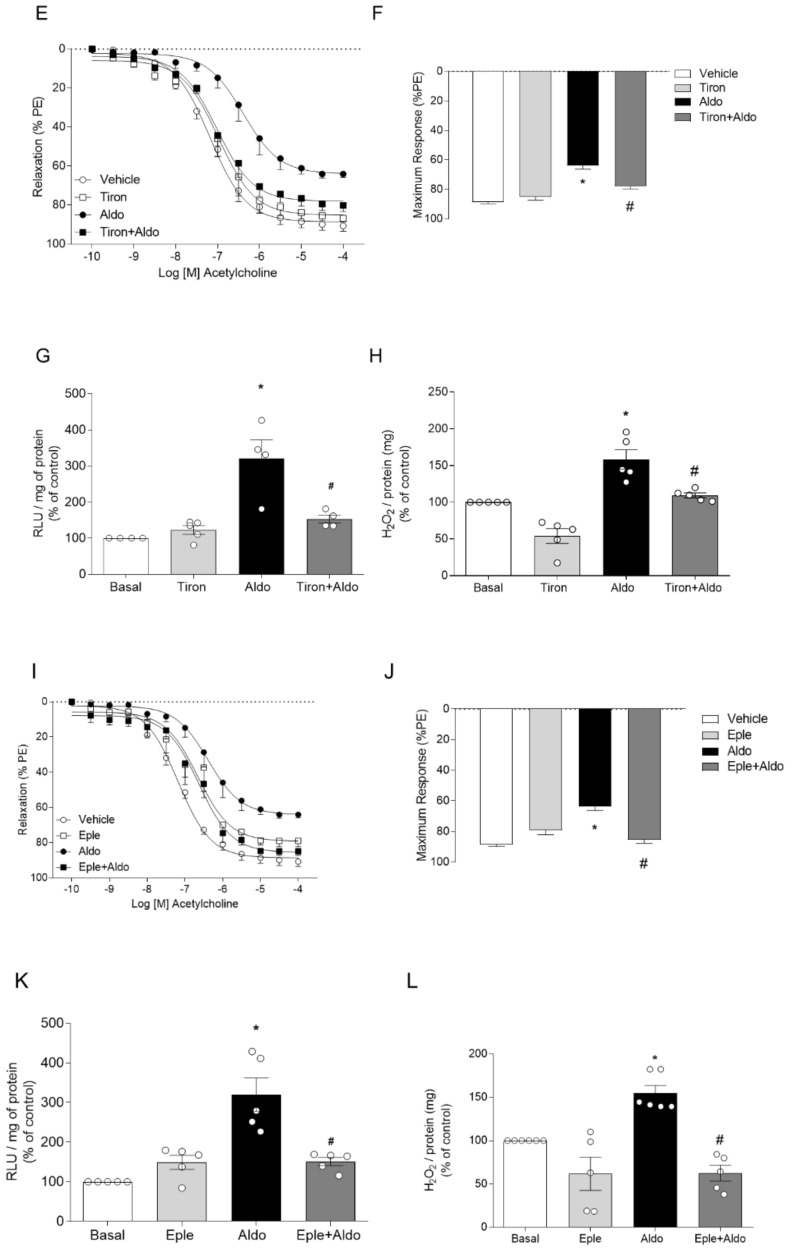
Aldosterone, via MR activation, induces vascular dysfunction and oxidative stress. Concentration-effect curves (**A**,**E**,**I**) and maximum responses (**B**,**F**,**J**) to acetylcholine in the thoracic aortas of male C57BL/6J mice. Superoxide anion (**C**,**G**,**K**) and hydrogen peroxide (**D**,**H**,**L**) generation. Experiments were performed in the presence of Aldo (100 nM for 30 min) plus vehicle, tiron (ROS scavenger, 100 μM for 30 min), or Eple (MR antagonist, 1 μM for 30 min). Data represent the mean ± SEM. One-way ANOVA: *, *p* < 0.05 vs. basal; #, *p* <0.05 vs. Aldo; *n* = 5–9 for each experimental group.

Vascular dysfunction induced by Aldo was determined in the thoracic aortic segments of C57BL/6J male mice (ex vivo approach). Aldo treatment (100 nM for 30 min) decreased endothelium-dependent vasorelaxation (Figure 1A,B). Aldo (100 nM for 30 min) also increased O_2_^−^ and H_2_O_2_ generation in isolated aortas (Figure 1C,D), as determined by lucigenin chemiluminescence and Amplex Red, respectively.

To determine whether Aldo induces vascular dysfunction via ROS generation, vascular function was assessed in thoracic aortic segments pretreated with tiron. Tiron (100 μM for 30 min) prevented Aldo-induced, impaired, endothelium-dependent vasorelaxation (Figure 1E,F) as well as Aldo-induced ROS generation (Figure 1G,H).

To address whether Aldo induces its vascular effects by MR activation, vascular function was assessed in thoracic aortic rings pretreated with eplerenone (Eple). The MR antagonist (1 μM for 30 min) prevented Aldo-induced, impaired vasorelaxation (Figure 1I,J, Table 1) and ROS generation (Figure 1K,L), which demonstrates that Aldo induces ROS generation by MR-dependent mechanisms.

### 2.2. Pharmacological Activation of Nrf2 Prevents Aldosterone-Induced Vascular Dysfunction and Oxidative Stress

**Figure 2:**
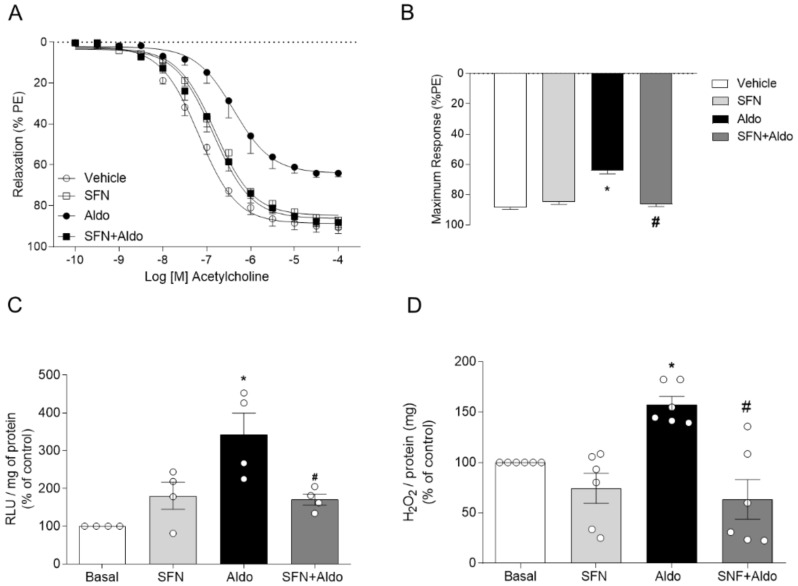
Pharmacological activation of Nrf2 attenuates aldosterone-induced impairment in redox state. Concentration-effect curves (**A**) and maximum response (**B**) to acetylcholine in the thoracic aortas of C57BL/6J mice. Superoxide anion (**C**) and hydrogen peroxide (**D**) generation. Experiments were performed in the presence of Aldo (100 nM for 30 min) or SFN (1 μM for 3 h). Data represent the mean ± SEM. One-way ANOVA: * *p* < 0.05 vs. basal; # *p* < 0.05 vs. Aldo; *n* = 5–9 for each experimental group.

To assess whether pharmacological activation of Nrf2 counteracts vascular effects induced by Aldo, functional studies were performed in aortic rings pretreated with L-sulforaphane (SFN), an Nrf2 activator. Aortic rings pretreated with SFN (1 μM for 3 h) did not display impaired vasorelaxation in response to Aldo (Figure 2A,B). Maximum responses (Emax) and pEC50 to ACh-induced vasorelaxation are presented in Table 2. SFN also prevented Aldo-induced vascular generation of ROS (Figure 2C,D).

### 2.3. Aldosterone Induces ROS Generation in Human Endothelial Cells 

**Figure 3:**
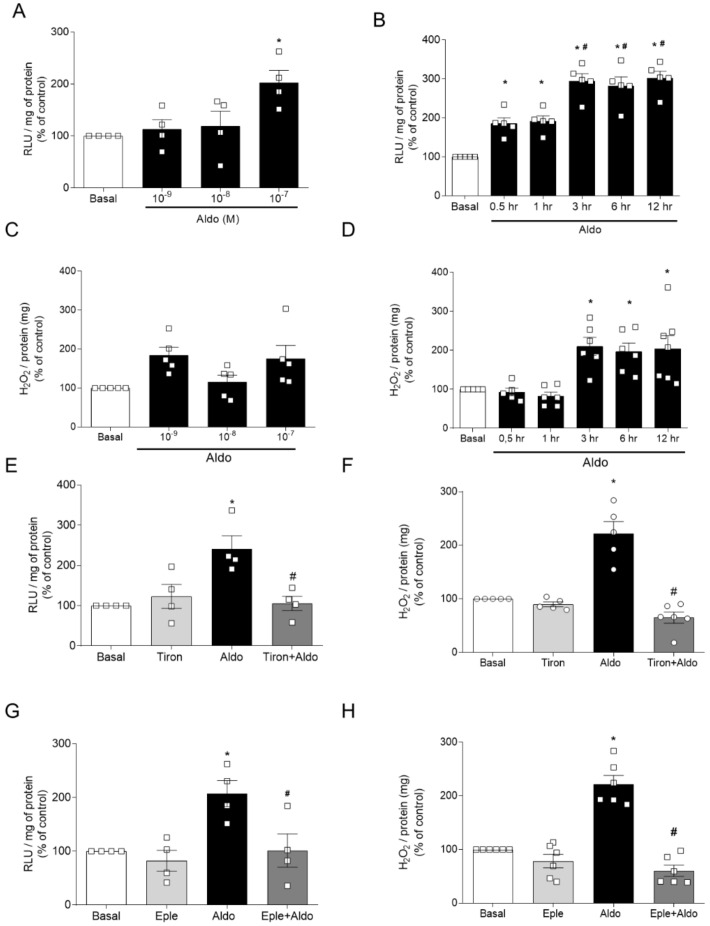

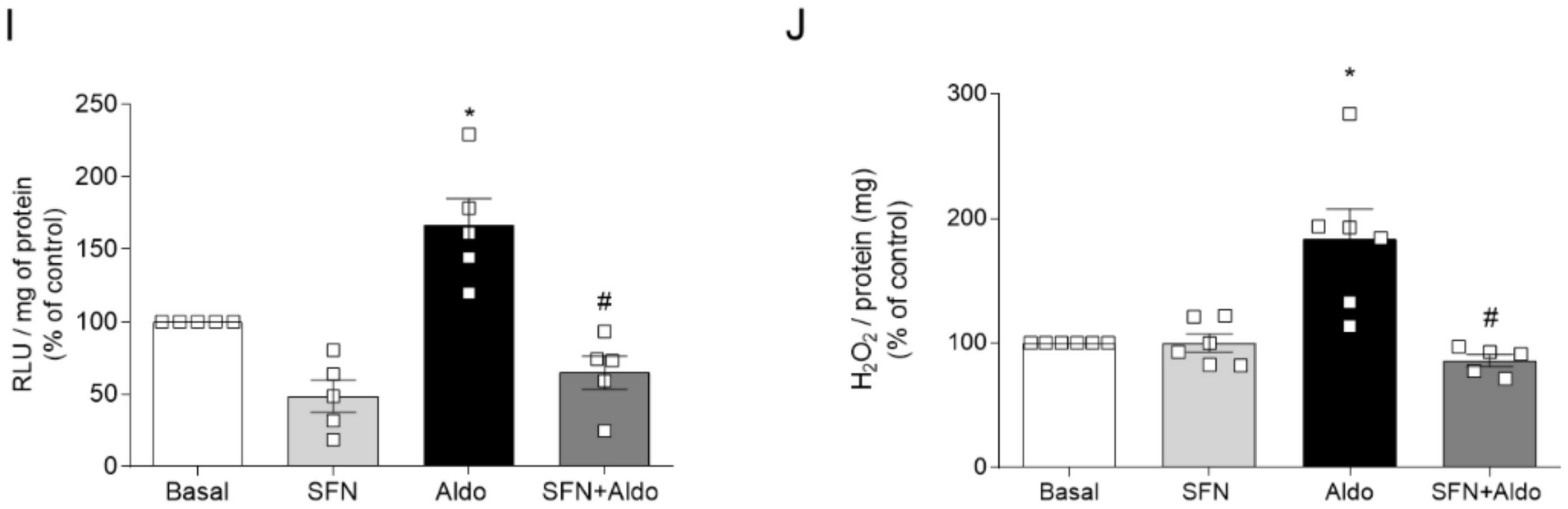
Aldosterone induces ROS generation in EA.hy926 cells. Superoxide anion (**A**,**B**) and hydrogen peroxide (**C**,**D**) generation in EA.hy926 cells treated with Aldo at various time points and concentrations. Superoxide anion (**E**,**G**,**I**) and hydrogen peroxide (**F**,**H**,**J**) generation in EA.hy926 cells treated with Aldo (100 nM for 30 min) in the presence of tiron (100 μM for 30 min), Eple (1 μM for 30 min), or SFN (1 μM for 3 h). Data represent the mean ± SEM. One-way ANOVA: * *p* < 0.05 vs. basal; #, *p* < 0.05 vs. 0.5 h. *n* = 4–6 for each experimental group.

To address mechanisms by which Aldo interferes with Nrf2 signaling, Aldo’s effects on ROS generation were determined in cultured human endothelial cells (EA.hy926). O_2_^−^ and H_2_O_2_ levels were measured after treatment of EA.hy926 cells with various concentrations of Aldo for 30 min. Aldo 100 nM for 30 min increased O_2_^−^ (Figure 3A), but not H_2_O_2_ (Figure 3C) levels. The time course for ROS generation was determined with 100 nM of Aldo. Aldo-induced O_2_^−^ generation increased after 30 min, and further increased after 3 h, a time point where H_2_O_2_ levels were also increased (Figure 3B,D). ROS levels were continuously sustained up to 6 h, suggesting that Aldo produces short- and long-term oxidative effects in human endothelial cells (Figure 3C,D).

Similar to Aldo’s effects on aortic rings, Aldo-induced ROS generation in EA.hy926 cells was prevented by an ROS scavenger, MR antagonist, and Nrf2 activator. Tiron (100 μM, 30 min) blocked O_2_^−^ and H_2_O_2_ generation (Figure 3E,F). Eplerenone (1 μM, 30 min), as well as pharmacological activation of Nrf2 with SFN (1 μM for 3 h), prevented Aldo-induced ROS generation in endothelial cells (Figure 3G–J, respectively).

### 2.4. p38 MAPK Inhibition Does Not Prevent Aldo-Induced Decreased Nrf2 Signaling 

**Figure 4:**
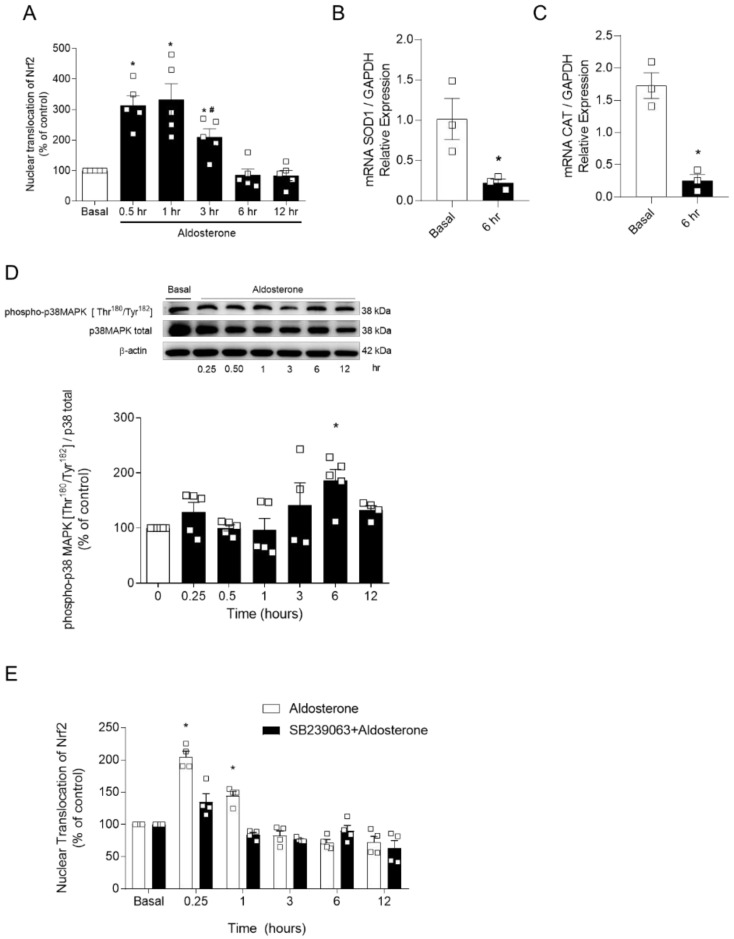
p38 MAPK inhibition does not prevent aldosterone-induced, reduced Nrf2 activity. Nuclear accumulation of Nrf2 (**A**) after treatment with Aldo and gene expression (**B**,**C**) of SOD1 and CAT. In (**D**), Aldo-induced p38 phosphorylation. (**E**), Nrf2 activity in the presence of vehicle or p38 MAPK inhibitor, SB239063 (50 μM). Data represent the mean ± SEM. One-way ANOVA: *, *p* < 0.05 vs. basal; #, *p* < 0.05 vs. 0.5 h. *n* = 3–5 for each experimental group.

To test the hypothesis that Aldo decreases Nrf2 activity, contributing to Aldo-induced oxidative stress, Nrf2 activity, determined by Nrf2 translocation assay, was assessed in cultured human endothelial cells. Figure 4A shows that Aldo activates Nrf2 in endothelial cells after 30 min. However, after 3 h, the nuclear accumulation of Nrf2 started to decrease compared to earlier time points (0.5 and 1 h). This was accompanied by reduced gene expression of Nrf2-regulated antioxidant enzymes, SOD1 (Figure 4B), and CAT (Figure 4C).

Considering that Aldo activates p38 MAPK, and that this kinase phosphorylates, among other targets, Keap1, we questioned whether Aldo-induced activation of p38 MAPK was responsible for Aldo-induced reduced Nrf2 activity. Initially, the time-course for Aldo-induced p38 phosphorylation was determined in cultured human endothelial cells (Figure 4D). Then, Nrf2 activity was measured in endothelial cells treated with Aldo in the presence of vehicle and SB239063, a selective p38 MAPK inhibitor, at different time points. As shown in Figure 4E, p38 MAPK inhibition did not prevent decreased Nrf2 accumulation induced by Aldo.

### 2.5. Aldosterone-Induced Reduced Nrf2 Activity Depends on the Expression of Nrf2-Negative Regulators

**Figure 5:**
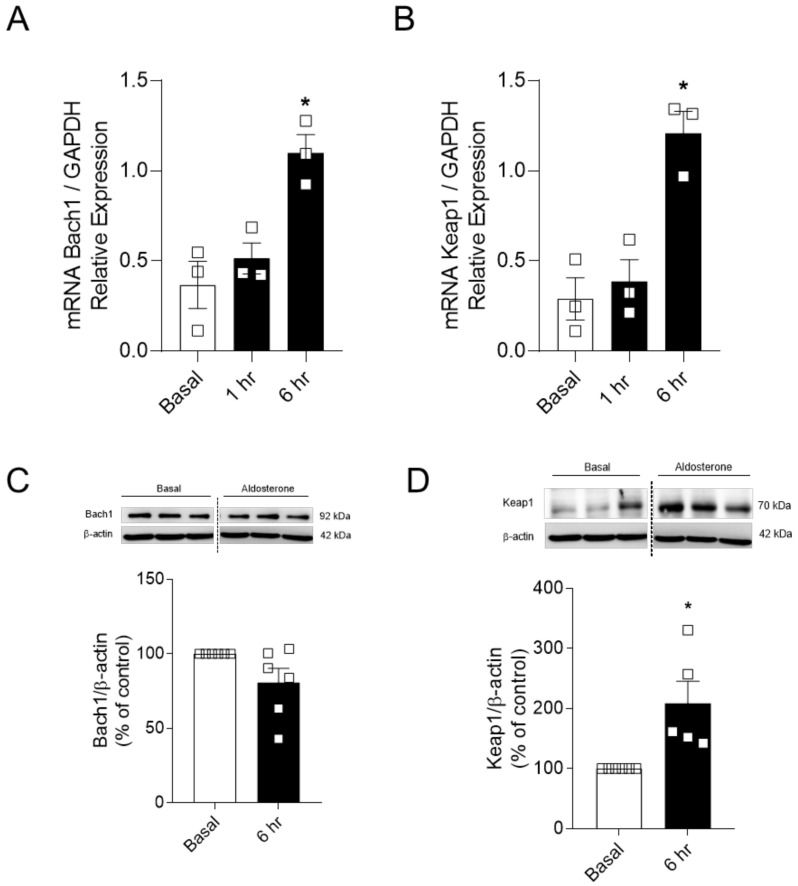
Aldosterone-induced reduced Nrf2 activity is linked to Keap1 upregulation. Gene and protein expression of Bach1 (**A**,**C**) and Keap1 (**B**,**D**). The vertical dotted line between the groups indicates that the image obtained from the same membrane was cropped. Data represent the mean ± SEM. One-way ANOVA: * *p* < 0.05 vs. basal. *n* = 3–6 for each experimental group.

Since p38 MAPK was not involved in reduced Nrf2 signaling induced by Aldo, the expression of critical Nrf2 repressor proteins, Keap1 and Bach1, was determined by RT-qPCR and Western blot. Using time points where Nrf2 activity was increased (1 h) and decreased (6 h), we observed that Aldo increased vascular protein expression of Keap1, but not Bach1 (Figure 5A–D).

### 2.6. In Vivo, Aldosterone Treatment Decreases Vascular Nrf2 Activity, and Increases Oxidative Stress and the Protein Expression of Keap1

**Figure 6:**
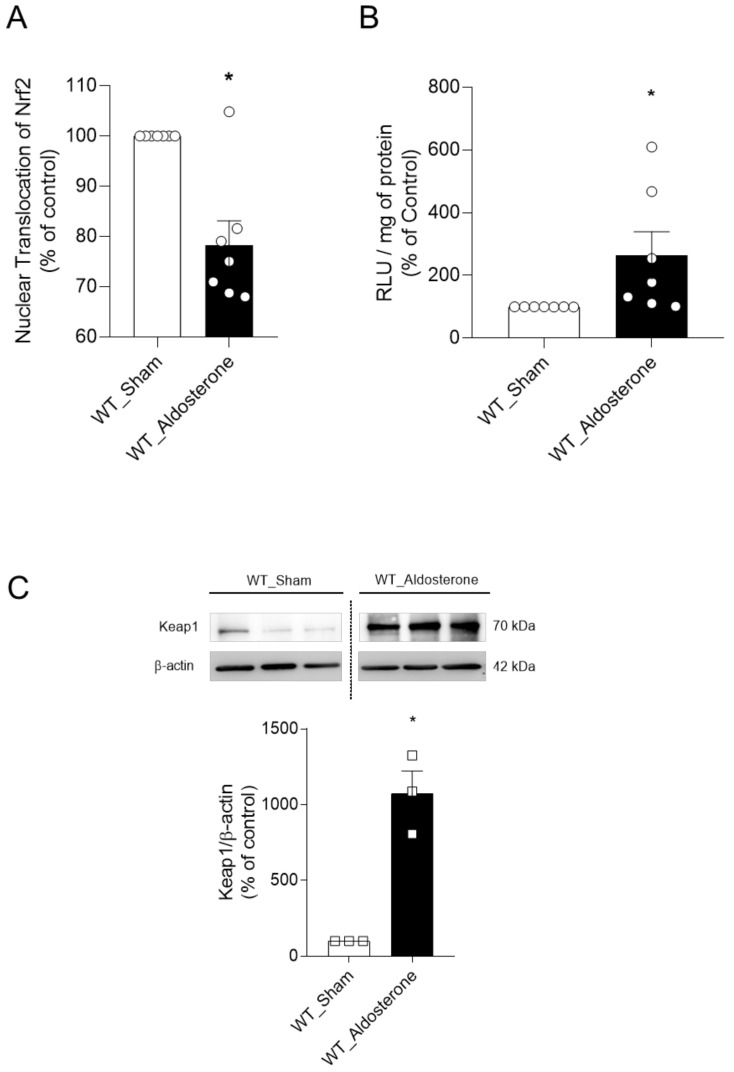
Aldosterone infusion in mice reduces vascular Nrf2 nuclear accumulation, increases Keap1 expression, and promotes oxidative stress. Nuclear accumulation of Nrf2 (**A**) and superoxide anion (**B**) levels in aortas from vehicle- and Aldo-infused mice. Protein expression of Keap1 (**C**) in aortas from vehicle- and Aldo-infused mice. The vertical dotted line between the groups indicates that the image obtained from the same membrane was cropped. Data represent the mean ± SEM. Student’s *t*-test: *, *p* < 0.05 vs. WT_Sham. *n* = 5–7 for each experimental group.

Isolated aortas from 12-week-old C57BL/6J male mice infused with Aldo (600 μg/kg per day) for 14 days exhibited reduced Nrf2 nuclear accumulation (Figure 6A) and increased oxidative stress (Figure 6B), as determined by the transAM Nrf2 ELISA kit and lucigenin chemiluminescence, respectively. Increased protein expression of the key negative regulator of Nrf2 signaling, Keap1, was found in arteries from Aldo-infused mice (Figure 6C).

## 3. Discussion

Our study shows that Aldo reduces the nuclear accumulation of Nrf2 by Keap1-dependent mechanisms. Aldo’s effects on Nrf2 signaling were identified in aortas incubated with Aldo (ex vivo), in aortas from Aldo-treated mice (in vivo), and in human endothelial cells (in vitro). Aldo induces two waves of ROS generation, the second of which is paralleled by decreased Nrf2 activity. Aldo-induced ROS generation and vascular dysfunction are prevented by the MR antagonist eplerenone, indicating that these events rely on MR activation. In addition, pharmacological activation of Nrf2 with L-sulforaphane abrogates Aldo-induced ROS generation and vascular dysfunction.

Aldo, a mineralocorticoid hormone isolated over 60 years ago, is vital for sodium handling by the kidneys. Aldo also produces vascular fibrosis, increases the expression of inflammatory markers, and induces oxidative stress.

High levels of Aldo increase cardiovascular risk. Subjects with hyperaldosteronism, as well as subjects on chronic treatment with angiotensin-converting enzyme (ACE) blockers or angiotensin type 1 receptor antagonists, exhibit increased Aldo levels, an event known as “aldosterone breakthrough” [28]. Aldo levels are also increased in obese subjects, and a close correlation between visceral adiposity and increased levels of Aldo, mainly in female subjects, has been reported [29].

Aldo induces oxidative stress, inflammation (that can ultimately lead to apoptosis), fibrosis, and metabolic effects, such as insulin resistance and adipocyte hypertrophy [6]. Accordingly, mineralocorticoid receptor antagonism lowers cardiovascular-related deaths by about 30%, as shown by many clinical trials [8,9].

The effects of Aldo are usually genomic, but rapid and non-genomic effects of Aldo, including oxidative stress by the activation of NOX enzymes and crosstalk with other systems—including the transactivation of angiotensin II type 1 receptor (AT1), G protein-coupled estrogen receptor 1 (GPER), and tyrosine kinase receptors—have also been reported [30]. For example, Aldo-induced ROS generation and renal fibrosis are linked to epidermal growth factor receptor (EGFR) activation [31]. GPER is required for non-genomic signaling by Aldo in vascular cells [32]. Furthermore, GPER activation seems to differentially contribute to Aldo-induced effects on blood pressure in males and females [33]. In general, these interactions of Aldo with other receptors are facilitated by localization in lipid-rich domains, also known as caveolae, culminating in protein kinase activation, amplification of downstream signaling, and increased ROS generation [30].

ROS have physiological roles [10,34,35]. However, when the generation of these volatile species is exacerbated, and cells are no longer able to handle them, the consequent malfunction of cells leads to and aggravates pathological conditions.

Our first aim was to address whether acute exposure of isolated aortas to Aldo induces vascular dysfunction and oxidative stress, and whether ROS generation induced by Aldo is responsible for vascular dysfunction (Figure 1 and Figure 2). Then, we determined whether MR mediates Aldo-induced vascular dysfunction and ROS generation (Figure 3). To investigate mechanisms involved in Aldo effects, we used cultured human umbilical endothelial cells (EA.hy926) and characterized the pattern of ROS generation induced by Aldo. Aldo induces ROS generation in a concentration- and time-dependent manner (Figure 3A–D). By using lucigenin chemiluminescence and Amplex Red assays, we confirmed that Aldo induces ROS generation by MR-dependent mechanisms in human endothelial cells (Figure 3E–H), similar to the observed in ex vivo experiments. As expected, pharmacological activation of Nrf2 in endothelial cells prevented increased ROS generation induced by Ado (Figure 3I,J).

The present data support previous studies showing that vascular dysfunction induced by Aldo is MR- and ROS-dependent [16,36,37,38]. ROS generation and MR activation contribute to Aldo-mediated vascular dysfunction in type 2 diabetes mellitus [39,40]. Of importance, the pharmacological activation of Nrf2 prevents endothelial dysfunction [41,42] and ameliorates redox parameters in chronic models of hypertension [43].

The contribution of Nrf2 signaling to counteract redox imbalance has been extensively studied in multiple diseases, including inflammatory [44], neurodegenerative [45], metabolic [46], and cardiovascular [47]. The first evidence showing the relationship between Aldo and Nrf2 was reported by Queisser et al. in 2014 [48] in porcine and rodent kidney cells, with Aldo activating Nrf2 in a concentration- and time-dependent manner. However, Nrf2 activation by Aldo in Queisser’s experiments failed to counteract long-term oxidative damage, suggesting that the antioxidant response induced by Nrf2 fails in conditions of chronic exposure to Aldo.

Therefore, we questioned whether Aldo reduces Nrf2 activity in endothelial cells (Figure 4A). Aldo treatment activated Nrf2, increasing its nuclear accumulation at short times. However, after 3 h of Aldo stimulation, Nrf2 nuclear accumulation decreased and reached basal levels between 6 and 12 h. Also, gene expression of antioxidant enzymes, such as SOD1 and catalase, decreased at the same time-points where Nrf2 activity was reduced (Figure 4B,C). These data indicate that chronic exposure to Aldo impairs Nrf2 signaling. Indeed, Nrf2 failure is found in models of chronic hypertension, inflammatory diseases, and ageing [46].

Protein kinases play a significant role in Nrf2 regulation and activity [22]. Considering that Aldo activates NOXes and p38 MAPK via c-Src [14], and p38 MAPK phosphorylates Keap1 and increases Keap1–Nrf2 interaction [49], we questioned whether p38 MAPK is involved in Aldo-induced Nrf2 failure. First, we confirmed that Aldo induces p38 MAPK phosphorylation (Figure 4D). Then, Nrf2 activity was assessed under Aldo stimulation in the presence of a p38 MAPK inhibitor, SB239063 (Figure 4E). However, p38 MAPK inhibition did not prevent Aldo-induced reduced Nrf2 activity.

Next, we questioned whether the negative regulators of Nrf2 signaling, Keap1 and Bach1, were involved in Aldo-induced decreased Nrf2 activity. Aldo increased both gene and protein expression of Keap1, but not Bach1, in endothelial cells (Figure 5). In vivo, Aldo infusion also increased vascular Keap1 expression (Figure 6), and this was accompanied by oxidative stress and reduced nuclear accumulation of Nrf2. Here, we speculate that increased vascular expression of Keap1 itself is linked to reduced Nrf2 activity in mineralocorticoid-induced vascular dysfunction. These findings reinforce that Nrf2 activator-based therapies can prevent these outcomes in chronic diseases where Nrf2 activity is impaired [43,50], once antioxidants such as resveratrol and SNF reduce Keap1 expression both in vitro and in vivo [51,52], allowing Nrf2 to translocate to the nucleus and start the antioxidant response.

## 4. Materials and Methods

### 4.1. Chemicals

Aldosterone, eplerenone, phenylephrine, and acetylcholine were purchased from Sigma-Aldrich (St. Louis, MO, USA). Tiron (Santa Cruz, Dallas, TX, USA), L-sulforaphane (Cayman, Ann Arbor, MI, USA), and SB 239063 (Tocris, Bristol, UK) were also purchased.

### 4.2. Animals

Twelve-week-old male C57BL/6J mice from the Animal Facility of the Ribeirao Preto Campus of the University of Sao Paulo were used. ALZET osmotic minipumps were implanted on mice for Aldo (600 μg/kg per day, for 14 days) or vehicle infusion into the extracellular space. For ex vivo vascular experiments, aortas were isolated from wild-type mice. The animals were housed in the Department of Pharmacology, Ribeirao Preto Medical School–USP, maintained in a room with controlled temperature (20 to 22 °C), 12 h light/dark cycles, 60% humidity, and food and water ad libitum.

### 4.3. Functional Vascular Studies

After anesthesia with isoflurane and euthanasia, thoracic aortas were isolated and placed in a dish containing a modified Krebs–Henseleit nutrient solution at 4 °C (composition in mM): NaCl = 130; KCl = 4.7; KH_2_PO_4_ = 1.18; MgSO_4_ = 1.17; NaHCO_3_ = 14.9; EDTA = 0.026; CaCl_2_ × 2H_2_O = 1.56; glucose = 5.5. The median portion of the thoracic aorta was divided into four rings of 2 mm each. Endothelium-intact rings were mounted on a myograph (model 620 M; Danish Myo Technology (DMT), Copenhagen, Denmark), and isometric force generation was recorded, using a signal transducer (ML T001 isometric voltage transducer, Power Lab/8S, AD Instruments Pty Ltd., Australia) coupled to a computer. The myograph vats contained modified Krebs–Henseleit solution, gassed with 95% O_2_ and 5% CO_2_, and heated to 37 °C. The preparations remained under 5 mN of tension for 30 min for stabilization, with changes in nutrient solution and tension adjustment every 10 min.

After the stabilization period, the integrity of the endothelium was tested by assessing relaxation to acetylcholine (ACh; endothelium-dependent vasodilator) in aortic rings pre-constricted with phenylephrine (PE; 10^−7^ M). The absence of relaxation to ACh or the presence of relaxation at up to 5% of pre-contraction levels was considered evidence of the successful removal of the endothelium. Concentration–effect curves to PE and ACh (10^−11^–10^−4^ M) were performed in endothelium-intact vascular segments. For each concentration–effect curve, the maximum response and pEC50 were calculated using non-linear regression analysis (GraphPad Prism Software).

### 4.4. Cultured Endothelial Cells

Immortalized human vascular endothelial cells (EA.hy926) were cultured in six-well plates, using Dulbecco’s Modified Eagle Medium (DMEM) supplemented with 10% of fetal bovine serum (FBS), and maintained in an incubator at 37 °C and 5% CO_2_. Once cells were at ~80% of confluence, they were stimulated with Aldo in the presence of vehicle, tiron, Eple, or SFN. After stimulation, cells were washed and harvested in lysis buffer and kept frozen until further use.

### 4.5. Lucigenin-Enhanced Chemiluminescence

Isolated thoracic aortas and endothelial cells (EA.hy926) were treated with Aldo (100 nM) in the presence of vehicle, tiron (ROS scavenger; 100 μM), eplerenone (1 μM), or L-sulforaphane (1 μM). After stimulation, cells/aortas were washed and harvested in ROS/lysis buffer. NADPH (10^−4^ mol/L) was added to the suspension containing lucigenin (5 μM). Luminescence was measured before and after stimulation with NADPH. A buffer blank was subtracted from each reading. The results are expressed as fold change in arbitrary units per milligram of protein (measured by the BCA assay).

### 4.6. Amplex Red

Measurement of vascular hydrogen peroxide (H_2_O_2_) levels was performed using the fluorescence Amplex Red Hydrogen Peroxide/Peroxidase Assay Kit (Molecular Probes), according to the manufacturer’s instructions. Cellular protein levels, measured by bicinchoninic acid assay (BCA assay), were used to normalize H_2_O_2_ production. The results are expressed in arbitrary units per milligram protein.

### 4.7. Nrf2 Translocation Assay

To determine the nuclear accumulation of Nrf2, samples were prepared according to the manufacturer’s protocol using the nuclear extract kit (Active Motif). The TransAM Nrf2 ELISA kit (Active Motif, Carlsbad, CA, USA) was used to quantify Nrf2 in nuclear preparations (10 μg), with absorbance values read at 450 nm of the wavelength.

### 4.8. Gene Expression Analysis by RT-qPCR

RNA was isolated from endothelial cells using Trizol (Invitrogen, Carlsbad, CA, USA). cDNA was synthesized using a High-Capacity cDNA Reverse Transcription Kit (Applied Biosystems), according to the manufacturer instructions. The mRNA levels were quantified on qPCR StepOnePlus (Life Technologies). Specific primers (TaqMan) for humans were used respectively as follows: SOD1 (Hs00533490_m1) CAT (Hs00156308_m1), Keap1 (Hs00202227_m1), Bach1 (Hs00230917_m1), and GAPDH (Hs_02786624_g1), all purchased from Life Technologies. Specific mRNA expression levels were normalized relative to GAPDH mRNA levels using the comparative 2^−ΔΔCt^ method.

### 4.9. Western Blot

Protein expression of p38 MAPK (total and phosphorylated), Keap1, Bach1, and beta-actin was determined by Western blot analysis in cultured endothelial cells and isolated aortas. Samples were homogenized in lysis buffer, and proteins were kept frozen in −80 °C until use. Proteins (30 μg) were separated by electrophoresis on 10% or 12% polyacrylamide gels, transferred to 0.22 μm nitrocellulose membranes, and blocked using 5% bovine serum albumin (BSA) in Tris-buffered saline (TBS) and 0.1% Tween 20 for 1 h. Primary antibodies were incubated overnight at 4 °C, as follows: anti-phospho p38 (phospho Thr180 + Tyr182) (1:1000; Cell Signaling 4511), anti-p38 (1:1000; Cell Signaling 9212), anti-Keap1 (1:1000; Abcam 66620), anti-Bach1 (1:1000; sc-271211), and anti-β-actin-peroxidase (1:10,000 dilution; Sigma-Aldrich). Protein bands were detected by chemiluminescence reaction (Luminata Forte, WBLUF0100, Merck-Millipore, Watford, UK), and the intensity of the bands was evaluated by densitometric analysis using ImageQuant software.

### 4.10. Statistical Analysis

For analysis of vascular reactivity, maximum response, and pEC50 (negative logarithm of EC50), the values were determined from the concentration–response curves. Emax and pEC50 values were compared using Student’s *t*-test and a one-way analysis of variance test (one-way ANOVA), followed by the Tukey post-test. The results of the molecular experiments were analyzed by Student’s *t*-test or one-way ANOVA, followed by the Tukey post-test. GraphPad Prism program, version 8.0 (GraphPad Software Inc., San Diego, CA, USA) was used for data analysis. The results are expressed as mean ± standard error of the mean (SEM). The acceptable level of significance was *p* < 0.05.

## 5. Conclusions

In conclusion, our study shows that Aldo negatively regulates Nrf2 activity through increased expression of its repressor Keap1, leading to worsening of the redox status and vascular dysfunction. These data support MR antagonism and Nrf2 activation as novel and promising candidates to ameliorate redox status in diseases associated with high aldosterone levels.

## Figures and Tables

**Table 1:** pEC50 values for ACh-induced relaxation in the thoracic aortas of C57BL/6J treated with aldosterone in the presence of vehicle, tiron, or eplerenone.

Vehicle	Tiron	Eple	Aldosterone	Tiron + Aldosterone	Eple + Aldosterone
7.18 ± 0.04	6.99 ± 0.08	6.66 ± 0.11	6.38 ± 0.10 ^*^	7.00 ± 0.07 ^#^	6.63 ± 0.08

Data represent the mean ± SEM. One-way ANOVA: * *p* < 0.05 vs. vehicle; # *p* < 0.05 vs. aldosterone; *n* = 5–9 for each experimental group. pEC50: negative logarithm of the EC50, Ach: acetylcholine.

**Table 2:** pEC50 values for ACh-induced relaxation in the thoracic aortas of C57BL/6J treated with aldosterone in the presence of vehicle or SFN.

Vehicle	SFN	Aldosterone	SFN + Aldosterone
7.18 ± 0.04	6.80 ± 0.06	6.38 ± 0.10 ^*^	6.85 ± 0.05 ^#^

Data represent the mean ± SEM. One-way ANOVA: * *p* < 0.05 vs. vehicle; ^#^ *p* < 0.05 vs. aldosterone. *n* = 5–9 for each experimental group. SFN: L-sulforaphane, pEC50: negative logarithm of the EC50, Ach: acetylcholine.

## Abbreviations

ACh	Acetylcholine
Aldo	Aldosterone
ARE	Antioxidant response element
BM	Bardoxolone methyl
BSA	Bovine serum albumin
Cul3	Cullin 3
Eple	Eplerenone
H_2_O_2_	Hydrogen peroxide
Keap1	Kelch-like ECH-associated protein 1
MR	Mineralocorticoid receptor
NO	Nitric oxide
ONOO-	Peroxynitrite
Nrf2	Nuclear factor erythroid 2-related factor 2
O2·-	Superoxide anion
OS	Oxidative stress
PA	Primary aldosteronism
pEC50	Negative logarithm of the EC50
PE	Phenylephrine
RLU	Relative luminescence units
ROS	Reactive oxygen species
SEM	Standard error of the mean
siRNA	Small interfering RNA
SFN	L-Sulforaphane
TBS	Tris-buffered saline
Vehicle	Ethanol
VSMC	Vascular smooth muscle cell
